# The impact of pullback measurement on treatment decision in significant coronary artery disease: Insights from a retrospective multicentric study

**DOI:** 10.1016/j.ijcha.2026.101887

**Published:** 2026-02-10

**Authors:** Roberto Bova, Matteo Betti, Samuel Heuts, Pieter A. Vriesendorp, Alexander J.J. Ijsselmuiden, Saman Rasoul, Mustafa Ilhan, Jindra Vainer, Ralph A.L.J. Theunissen, Leo F. Veenstra, Patty Winkler, Mera Stein, Alexander Ruiters, Daniek P.J. Slegers, Arnoud W.J. van ’t Hof, Arpad Lux

**Affiliations:** a Department of Cardiology, Maastricht University Medical Centre+, Maastricht, The Netherlands; b Cardiovascular Research Institute Maastricht, Maastricht, The Netherlands; c Department of Clinical Sciences and Community Health, Cardiovascular Section, University of Milan, Milan, Italy; d Department of Cardiothoracic surgery, Maastricht University Medical Centre+, Maastricht, The Netherlands; e Department of Cardiology, Zuyderland Medical Center, Heerlen, The Netherlands

**Keywords:** Coronary physiology, Pullback, Revascularization, Optimal medical therapy

## Abstract

**Background:**

Optimal management of coronary artery disease (CAD) requires tailoring treatment strategies to lesion characteristics. Intracoronary pullback enables hemodynamic mapping of coronary lesions, potentially improving therapeutic decision-making, particularly in distinguishing focal from diffuse disease.

**Objectives:**

To evaluate how pullback measurement influences overall treatment strategy—optimal medical therapy (OMT), percutaneous coronary intervention (PCI), or coronary artery bypass grafting (CABG)—in patients with significant CAD.

**Methods:**

We conducted a retrospective, multicenter cohort study including 842 patients with stable angina, unstable angina, or non-ST-elevation myocardial infarction (NSTEMI) and functionally significant left anterior descending artery (LAD) disease. Patients were stratified into two groups: one group (PB group, n = 561) had pullback measurement, and the other (Conventional group, n = 281) not. Outcomes included treatment strategy, major adverse cardiovascular events (MACE), and all-cause mortality at 1 year.

**Results:**

Pullback led to more Heart Team discussions (66.3% vs. 58.7%; p = 0.033), greater adoption of OMT (51.5% vs. 40.9%; p = 0.004), and lower PCI rates (27.1% vs. 36.3%; p = 0.007). CABG rates remained unaffected. Pullback independently increased the odds of OMT and reduced the odds of PCI (OR = 0.58, p = 0.003), while three-vessel disease strongly predicted CABG (OR = 2.51; p < 0.001). At 1 year, the PB group had higher mortality (4.3% vs. 1.1%, p = 0.013), but similar MACE compared to the Conventional group. However, clinical outcomes did not differ between treatment groups.

**Conclusions:**

Intracoronary pullback favours a conservative treatment strategy. MACE rates are not increased at 1 year.

## Graphical abstract

**Figure:**
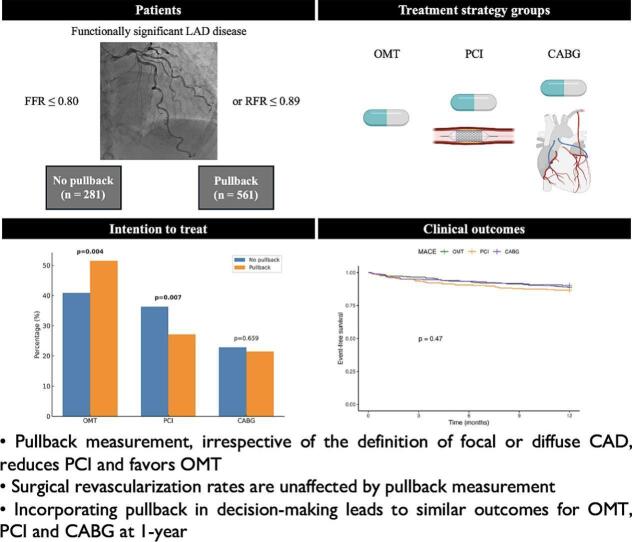

## 1 Introduction

The assessment of coronary artery disease (CAD) severity has significantly evolved, transitioning from purely angiographic evaluations toward ischemia testing and physiologic measurements to guide clinical decisions [1], [2], [3], [4]. While initially validated in chronic coronary syndromes (CCS), physiologic assessment has also been applied in acute coronary syndromes (ACS), particularly in deciding the timing and extent of revascularization of non-culprit lesions thus reducing unnecessary interventions and enhancing clinical outcomes [5], [6].

Pullback measurement techniques—whether hyperaemic (i.e., Fractional Flow Reserve [FFR]) or non-hyperaemic—enhance the granularity of coronary assessment by mapping the physiological significance of lesions along the coronary artery. While a common angiographic criterion to distinguish focal from diffuse lesions is to consider a length-cutoff of 20 mm, pullback techniques differentiate according to the distribution and magnitude of pressure losses along the vessel [7], [8]. Typically, discordance between the two modalities is encountered in the presence of diffuse coronary artery disease. This disease phenotype is characterized by a progressive pressure decrease without a focal drop. Importantly, pullback allows reclassification of the disease pattern in more than one third of cases compared to visual estimation [9].

Evidence from Collet et al. highlights that focal lesions respond optimally to PCI due to precise targeting of the stenotic segment, significantly improving post-interventional physiological measurements and patient outcomes [10]. On the other hand, diffuse disease is associated with suboptimal post-PCI physiological results which has been repeatedly associated with vessel-oriented adverse outcomes and residual angina at follow-up [11], [12]. Consequently, diffuse lesions are primarily managed with optimal medical treatment (OMT) with or without surgical revascularization. However, the preferred method of treatment and its influence on clinical outcomes are unknown. We performed this analysis to delineate the precise effect of pullback measurement on treatment selection.

## 2 Methods

### 2.1 Study design and population

This retrospective cohort study was approved by the local Medical Ethical Committee (METC) on 04/10/2022 (number 2022–3303) and was conducted at two medical centres: Zuyderland Medical Center (Heerlen, the Netherlands) and Maastricht University Medical Center+ (Maastricht, the Netherlands). The study population comprised patients aged ≥ 18 years diagnosed with stable angina, unstable angina or non-ST-elevation myocardial infarction (NSTEMI), who underwent coronary angiography and had a positive flow reserve assessment—defined as FFR ≤ 0.80 or resting full-cycle ratio (RFR) ≤ 0.89—in the left anterior descending (LAD) artery, between January 1, 2022, and January 1, 2024. Exclusion criteria were as follows: recent ST-elevation myocardial infarction (STEMI) within 30 days prior to coronary angiogram, history of in-stent restenosis, history of coronary artery bypass grafting (CABG), and presence of left main coronary artery disease.

All included patients were followed for 1 year to evaluate the occurrence of all cause-mortality and major adverse cardiovascular events (MACE, a composite of cardiovascular mortality, myocardial infarction, urgent revascularization and stroke).

### 2.2 Data collection

Clinical data were retrieved from the electronic patient records. Baseline characteristics were extracted from the prospectively collected Heart Team reports and coronary angiography reports and images, with follow-up collected through a 1-year period. All outcomes were adjudicated based on source documentation available in the electronic health records.

### 2.3 Intracoronary measurements

FFR and RFR measurements were performed using the PressureWire™ X Guidewire system in conjunction with the CoroFlow‡ Cardiovascular System (Abbott Cardiovascular) [2], [13]. RFR was measured during manual wire pullback to obtain a continuous assessment of pressure changes along the vessel. For the purposes of this study, only positive measurements in the LAD were included—defined as FFR ≤ 0.80 or RFR ≤ 0.89. Patients with a documented pullback procedure were categorized into the Pullback group (PB), while those without were assigned to the No pullback group (Conventional).

### 2.4 Statistical analysis

Descriptive statistics were used to summarize and present baseline characteristics for both the Pullback (PB) and No pullback (Conventional) groups. Continuous variables were reported as means with standard deviations or as medians with interquartile ranges, depending on their distribution. Distributions were assessed visually (P-P plots) and tested statistically by Kolmogorov-Smirnov's test. Categorical variables were presented as absolute counts and corresponding percentages.

To address missing data, a pairwise deletion approach was applied: analyses were conducted using all available data for each specific comparison, while missing values in unrelated variables were excluded. The variables affected by missing data included EuroSCORE II, body mass index (BMI), left ventricular ejection fraction (LVEF), dyspnoea, angina pectoris, chronic obstructive pulmonary disease, cerebrovascular accident, hypercholesterolemia, pulmonary hypertension, peripheral artery disease, and stable CAD in medical history. Of note, all angiographic data and outcomes were 100% complete.

Comparative analyses between the PB and Conventional groups were conducted using appropriate statistical tests. Continuous variables were analysed using either Student's *t*-test or the Mann–Whitney *U* test, depending on the data distribution. Categorical variables were compared using the Chi-square test or Fisher's exact test (in case of cell count < 5) as appropriate.

The primary outcome of the study was the treatment strategy, which could range from OMT, to PCI, to CABG. To adjust for potential confounders, a multinomial regression analysis was performed to evaluate the association between baseline variables, coronary characteristics, and eventual treatment strategy. Consequently, the eventual effect was expressed in an odds ratio (OR) with 95% confidence interval (CI) for PCI or CABG, relative to OMT (i.e., OMT as the reference category).

Adverse events—all-cause mortality and MACE—were reported as observed number of events and as Kaplan–Meier estimated rates. Event-free survival up to one year was evaluated according to the unadjusted Kaplan–Meier method and survivals among subgroups were compared using the log-rank test. Patients without an event within this time frame were censored.

All statistical analyses were performed using R (version 4.4.2, R Foundation for Statistical Computing, Vienna, Austria) and IBM SPSS Statistics, version 28 (IBM Corp., Armonk, NY, USA).

## 3 Results

### 3.1 Patient demographics and characteristics

**Fig. 1:**
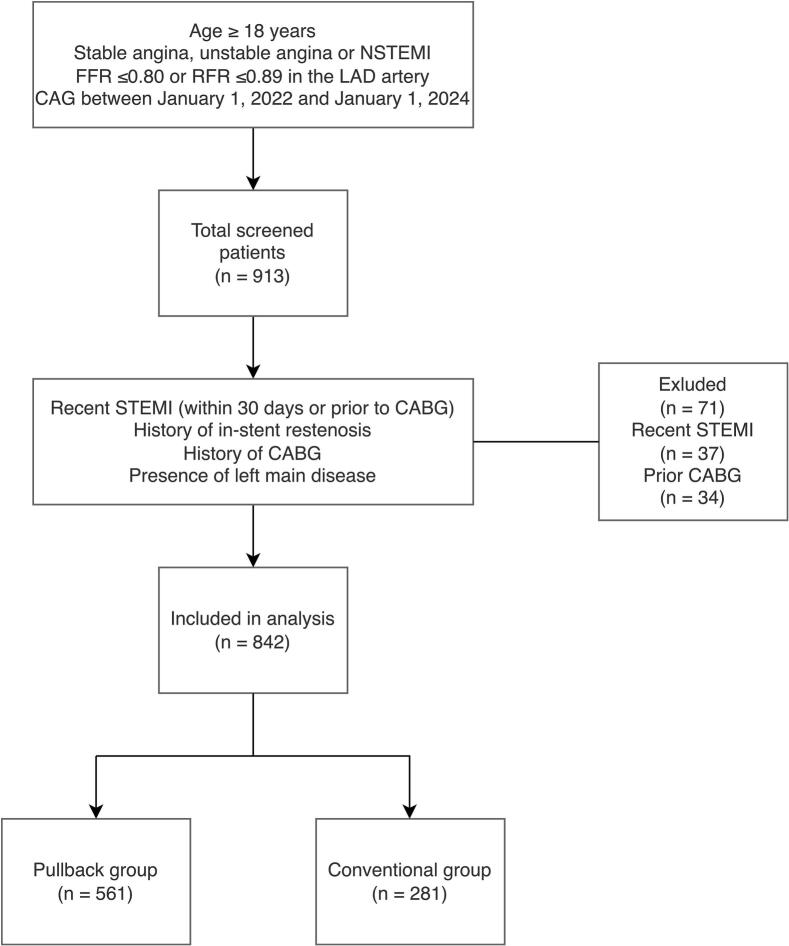
**Flowchart illustrating the inclusion process in the study**. CABG = coronary artery bypass grafting, CAG = coronary angiogram, FFR = Fractional Flow Reserve, LAD = left anterior descending, NSTEMI = non-ST-elevation myocardial infarction, RFR = Resting Full-Cycle Ratio, STEMI = ST-elevation myocardial infarction.

From the total of 913 patients screened, 37 were excluded due to recent STEMI, while 34 were excluded because of prior CABG (Fig. 1). A total of 842 patients were included in the analysis, of whom 561 (66.6%) belonged to the Pullback group (PB) and 281 (33.4%) to the No pullback group (Conventional).

Baseline characteristics are reported in Table 1. There were no statistically significant differences between the groups regarding most cardiovascular risk factors, including age (70 [63–76] vs. 68 [62–75] years; p = 0.071), diabetes mellitus (26.9% vs 21.7%, p = 0.106), hypertension (36.7% vs 74.4%, p = 0.536) and hypercholesterolemia (65.8% vs 66.6%, p = 0.764). LVEF (56% [49.5–60.5] vs 57% [45–60], p = 0.76) and BMI (26.7 [24.6–29.6] vs 27 [24.0–31.8], p = 0.757) did not differ between groups, as well as chronic obstructive pulmonary disease, cerebrovascular accident, peripheral artery disease and smoking status.

**Table 1:** Baseline characteristics.

	**No pullback (n = 281)**	**Pullback (n = 561)**	**p-value**
Age	70 (63–76)	68 (62–75)	0.071
Male sex	223 (79.4%)	452 (80.6%)	0.678
Diabetes	61 (21.7%)	151 (26.9%)	0.106
Hypertension	209 (74.4%)	206 (36.7%)	0.536
Hypercholesterolemia	187 (66.6%)	369 (65.8%)	0.764
COPD	34 (12.1%)	61 (10.9%)	0.289
CVA	34 (12.1%)	71 (12.7%)	0.818
PAD	27 (9.6%)	54 (9.6%)	0.994
PH	7 (2.5%)	25 (4.5%)	0.244
Dyspnoea	97 (34.5%)	219 (39%)	0.795
Angina pectoris	121 (43.1%)	281 (50.1%)	0.432
Smoking	70 (24.9%)	112 (20%)	0.1
Prior ACS	37 (13.2%)	108 (19.3%)	**0.044**
Known CAD	53 (18.9%)	136 (24.2%)	0.643
3-vessel disease	71 (25.3%)	140 (25%)	0.922
EuroSCORE II	1.3 (0.9–1.8)	1.1 (0.8–1.7)	0.106
BMI	27 (24–31.8)	26.7 (24.6–29.6)	0.757
LVEF (%)	57 (45–60)	56 (49.5–60.5)	0.76
Creatinine	92 (80–110)	91 (82.5–102.5)	0.96
eGFR	70.4 (56.5–80.9)	71.8 (61–81.5)	0.46
LDL	2.5 (1.8–3.4)	2.3 (1.9–3.2)	0.28
HDL	1.2 (1–1.6)	1.2 (1–1.4)	0.75
Total cholesterol	4.3 (3.6–5.5)	4.2 (3.6–5.4)	0.34
Haemoglobin	9.3 (8.6–9.7)	8,7 (8–9.3)	0.73
HbA1c	42.5 (39–50)	48 (40–62)	**0.01**

ACS = acute coronary syndrome, BMI = body mass index, CAD = coronary artery disease, COPD = chronic obstructive pulmonary disease, CVA = cerebrovascular accident, eGFR = estimated glomerular filtration rate, HbA1c = glycated haemoglobin, HDL = high-density lipoprotein cholesterol, LDL = low-density lipoprotein cholesterol, LVEF = left ventricular ejection fraction, PAD = peripheral artery disease, PH = pulmonary hypertension.

The only two important differences in baseline characteristics were prior ACS which was significantly higher in the PB group (19.3% vs 13.2%, p = 0.044) and HbA1c which was also significantly higher in the PB group (48 [40–62]) vs 42.5 [39–50], p = 0.01).

Angiographic LAD anatomy did not differ significantly between the Pullback and Conventional groups (Supplementary Table 1).

### 3.2 Treatment selection

Patients who underwent pullback measurement were more frequently discussed in the Heart Team (66.3% vs 58.7%, p = 0.033) (Table 2). Also, pullback measurement decreased the number of percutaneous interventions (27.1% vs. 36.3%, p = 0.007) and increased the proportion of patients treated with OMT (51.5% vs. 40.9%, p = 0.004). The rate of surgical revascularization was comparable.

**Table 2:** Treatment decision after CAG and according to the intention to treat principle, considering the decision after heart team discussion, in the Pullback and No pullback groups.

	**No pullback (n = 281)**	**Pullback (n = 561)**	**p-value**
Decision after CAG			
OMT	70 (24.9%)	128 (22.8%)	0.546
PCI	46 (16.4%)	61 (10.9%)	**0.028**
Heart Team	165 (58.7%)	372 (66.3%)	**0.033**
Intention to treat			
OMT	115 (40.9%)	289 (51.5%)	**0.004**
PCI	102 (36.3%)	152 (27.1%)	**0.007**
CABG	64 (22.8%)	120 (21.4%)	0.659

CABG = coronary artery bypass grafting, OMT = optimal medical therapy, PCI = percutaneous coronary intervention.

### 3.3 Clinical outcomes according to pullback measurement

At 1-year follow-up, the PB group exhibited a significantly higher all-cause mortality rate compared to the Conventional group (4.3% vs. 1.1%, p = 0.013), while no significant differences were observed in cardiovascular or non-cardiovascular mortality considered individually. MACE rates did not differ significantly between the two groups (11% vs. 12%; p = 0.709) (Table 3).

**Table 3:** Clinical outcomes in the Pullback and No Pullback groups.

	**No pullback (n = 281)**	**Pullback (n = 561)**	**p-value**
Mortality at 1 year	3 (1.1%)	24 (4.3%)	**0.013**
CV death	0 (0%)	8 (1.4%)	0.057
Non-CV death	3 (1.1%)	16 (2.9%)	0.1
MACE at 1 year	34 (12%)	63 (11%)	0.709
CV death	0 (0%)	8 (1.4%)	0.057
Myocardial infarction	15 (5.3%)	24 (4.3%)	0.49
Urgent revascularization	14 (5.0%)	23 (4.1%)	0.556
Stroke	5 (1.8%)	8 (1.4%)	0.769

CV = cardiovascular, MACE = major adverse cardiovascular events.

**Fig. 2:**
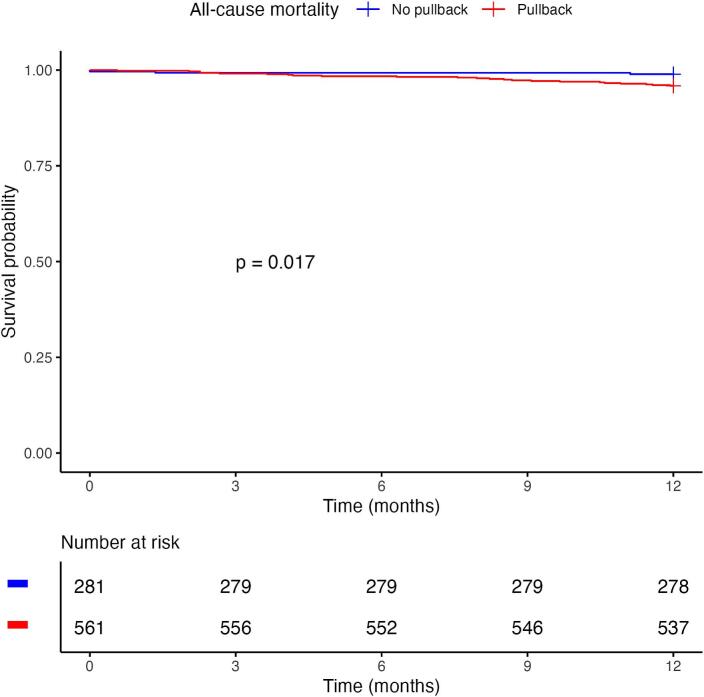

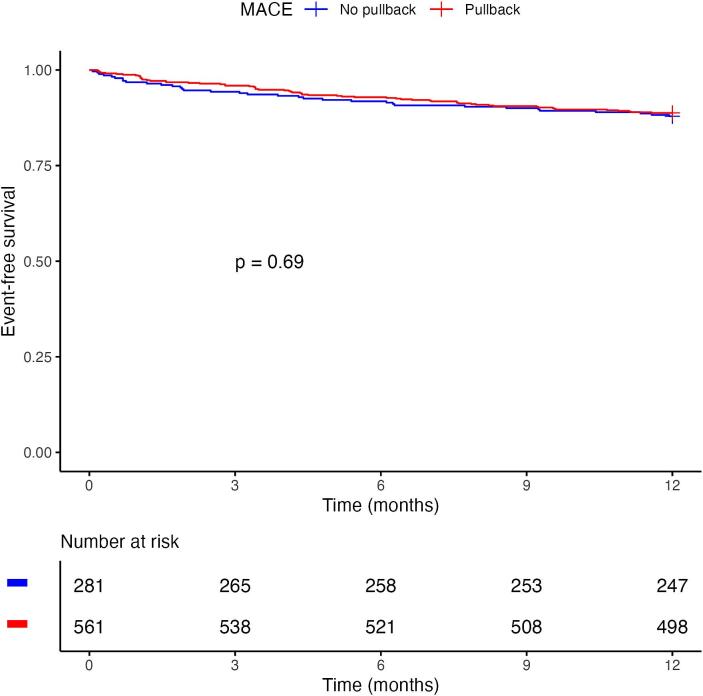
**a. kaplan–meier curves for all-cause mortality for patients in the pullback versus no pullback (conventional) group**. **b. Kaplan–Meier MACE-free survival curves for patients in the PB versus No pullback (Conventional) group**. MACE = major adverse cardiovascular events.

Fig. 2a shows the Kaplan–Meier survival curves for PB versus Conventional group. Restricted mean survival time at 1 year was 359 days (±1.6) for the Pullback group and 362 (±1.7) for the Conventional group (log-rank p = 0.017). Median survival time was not reached in either group.

Fig. 2b shows the Kaplan–Meier MACE-free survival curves for PB versus Conventional group. At 12 months, the estimated MACE-free survival was 88.8% (95% CI 86.2–91.4) in the PB group and 87.9% (95% CI 84.2–91.8) in the Conventional group (log-rank p = 0.69). Median MACE-free survival was not reached in either group.

One-year clinical outcomes in the Pullback cohort were comparable across treatment strategies (Supplementary Table 3).

### 3.4 Predictors of treatment decision

Across the 842 patients included in the study, 404 (48%) were managed with OMT, 254 (30.2%) with PCI and 184 (21.9%) with CABG. Baseline demographics and clinical characteristics are summarized in Supplemental Table 2. Of note, median age was lower in the CABG cohort while three-vessel disease was significantly more prevalent in patients referred to CABG.

To assess the influence of pullback measurement among other factors on treatment decision, multinomial logistic regression analyses were conducted. Variables were selected for inclusion in the model based on their clinical relevance. Our multivariable multinomial logistic regression shows that performing a pullback measurement increases the odds of OMT and lowers the odds of PCI (OR = 0.58, p = 0.003), as reported in Table 4. The analysis also highlights that three-vessel disease is the only strong independent predictor of CABG (OR = 2.51, p < 0.001). All other variables (sex, age, diabetes mellitus, chronic obstructive pulmonary disease, cerebrovascular accident, LVEF) were not statistically significant in the multivariable analysis.

**Table 4:** Univariable and multivariable multinomial logistic regression. Optimal medical therapy (OMT) is used as the reference category.

		**Univariable**			**Multivariable**		
		**OR**	**95% CI**	**p-value**	**OR**	**95% CI**	**p-value**
**Sex (male)**	*PCI*	1.30	0.88–1.92	0.192	1.31	0.86–2.00	0.205
	*CABG*	1.64	1.03–2.61	**0.036**	1.43	0.87–2.34	0.157
**Age (per year)**	*PCI*	1.00	0.98–1.02	0.847	1.00	0.98–1.02	0.792
	*CABG*	0.97	0.95–0.99	**0.004**	0.98	0.96, 1.00	0.051
**3VD**	*PCI*	0.98	0.67–1.45	0.921	0.94	0.62–1.42	0.760
	*CABG*	2.62	1.79–3.83	**<0.001**	2.51	1.66–3.80	**<0.001**
**DM**	*PCI*	1.17	0.82–1.68	0.391	1.13	0.76–1.68	0.535
	*CABG*	1.21	0.82–1.81	0.340	1.11	0.72–1.72	0.638
**COPD**	*PCI*	1.11	0.66–1.87	0.685	1.07	0.62–1.85	0.814
	*CABG*	1.08	0.60–1.92	0.800	1.06	0.57–1.98	0.856
**CVA**	*PCI*	1.28	0.82–2.02	0.278	1.39	0.86–2.25	0.179
	*CABG*	0.67	0.37–1.22	0.192	0.74	0.40–1.39	0.348
**LVEF**	*PCI*	0.99	0.98–1.01	0.449	1.0	0.98–1.01	0.518
	*CABG*	1.01	0.99–1.03	0.260	1.01	0.99–1.03	0.190
**Pullback**	*PCI*	0.59	0.43–0.83	**0.002**	0.58	0.41–0.83	**0.003**
	*CABG*	0.75	0.51–1.08	0.123	0.84	0.56–1.25	0.387

3VD = three-vessel disease, CABG = coronary artery bypass grafting, COPD = chronic obstructive pulmonary disease, CVA = cerebrovascular accident, DM = diabetes mellitus, LVEF = left ventricular ejection fraction, PCI = percutaneous coronary intervention.

### 3.5 Treatment strategy groups: clinical outcomes

Importantly, when considering the treatment strategy groups, 1-year mortality (OMT: 3.2% vs. PCI: 3.5% vs. CABG: 2.7%; p = 0.889) and MACE (11% vs. 13% vs. 9.8%; p = 0.479) did not differ significantly (Table 5).

**Table 5:** Clinical outcomes in the treatment strategy groups.

	**OMT (n = 404)**	**PCI (n = 254)**	**CABG (n = 184)**	**p-value**
Mortality at 1 year	13 (3.2%)	9 (3.5%)	5 (2.7%)	0.889
CV death	5 (1.2%)	2 (0.8%)	1 (0.5%)	0.813
Non-CV death	8 (2.0%)	7 (2.8%)	4 (2.2%)	0.793
MACE at 1 year	45 (11%)	34 (13%)	18 (9.8%)	0.479
CV death	5 (1.2%)	2 (0.8%)	1 (0.5%)	0.813
Myocardial infarction	14 (3.5%)	16 (6.3%)	9 (4.9%)	0.238
Urgent revascularization	19 (4.7%)	12 (4.7%)	6 (3.3%)	0.698
Stroke	7 (1.7%)	4 (1.6%)	2 (1.1%)	0.936

CABG = coronary artery bypass grafting, CV = cardiovascular, MACE = major adverse cardiovascular events, OMT = optimal medical therapy, PCI = percutaneous coronary intervention.

**Fig. 3:**
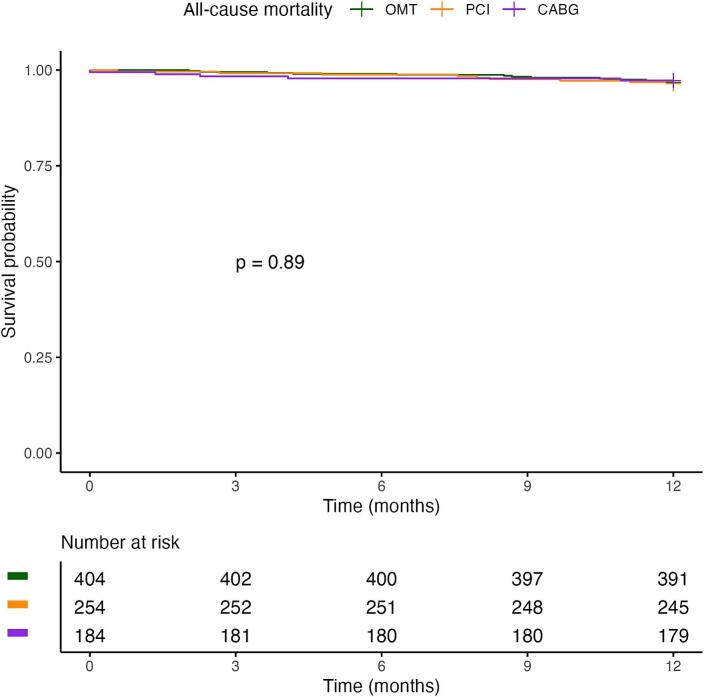

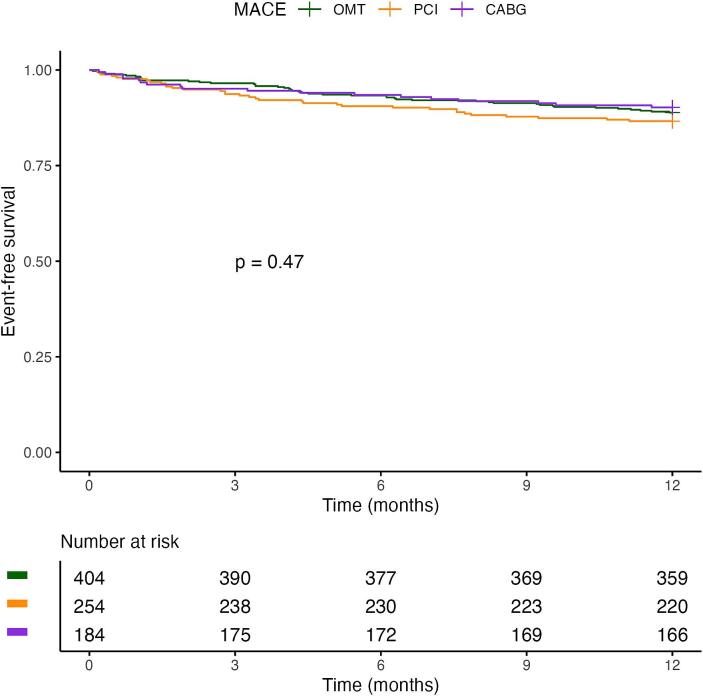
**a. kaplan–meier curves for all-cause mortality for patients in the three treatment strategy groups**. CABG = coronary artery bypass grafting, OMT = optimal medical therapy, PCI = percutaneous coronary intervention. **b. Kaplan–Meier MACE-free survival curves for patients in the three treatment strategy groups**. CABG = coronary artery bypass grafting, MACE = major adverse cardiovascular events, OMT = optimal medical therapy, PCI = percutaneous coronary intervention.

Fig. 3a shows the Kaplan–Meier survival curves for the treatment strategy groups. Restricted mean survival time at 1 year was 361 days (±1.5) for the OMT group, 360 (±2.1) for the PCI group and 358 (±3.3) for the CABG group (log-rank p = 0.89). Median survival time was not reached in either group.

Fig. 3b shows the Kaplan–Meier MACE-free survival curves for the treatment strategy groups. At 12 months, the estimated MACE-free survival was 88.9% (95% CI 85.8–92) in the OMT group, 86.6% (95% CI 82.5–90.9) in the PCI group and 90.2% (95% CI 86.0–94.6) in the CABG group (log-rank p = 0.47). Median MACE-free survival was not reached in either group.

## 4 Discussion

The current study investigated the impact of pullback measurement on treatment decision in a cohort of 842 patients with significant CAD involving the LAD artery. Our findings suggest that incorporating pullback measurement into routine practice promotes a conservative approach, primarily as an alternative to percutaneous procedures. Surgical revascularization is preferred in the presence of three-vessel disease, irrespective of pullback measurement.

Physiology-guided revascularization strategies reduce unnecessary interventions. Several trials have proven that this approach is more effective than the traditional angiography-guided one and, as a result, current guidelines recommend its implementation in our daily practice [1], [2], [3], [5], [6], [14], [15], [16]. The bulk of the data supporting the use of physiology to guide revascularization pertains to CCS. The DEFER trial demonstrated the safety of deferring PCI for lesions with an FFR ≥ 0.75 and the FAME study proved that FFR-guided PCI for lesions with FFR ≤ 0.80 can reduce MACE compared to angiography-guided PCI [1], [2]. These findings were further validated by FAME 2, which showed that using FFR to guide PCI decreased the need for urgent revascularization compared to using OMT only [3]. When considering ACS, current evidence supports the safety and efficacy of deferring PCI for lesions with negative FFR in the setting of NSTE-ACS [15]. Based on these data we decided to include both CCS and NSTE-ACS patients in our analysis. Aligning with the definitions of previous trials and current recommendations, we adopted as cut-off values for ischemia either an FFR ≤ 0.80 or an RFR ≤ 0.89.

After the initial coronary angiography, in 561 patients (66.6%) a pressure-wire pullback was obtained. All pullback measurements were performed at the time of coronary angiography, at the discretion of the operator, and were not demanded by the heart team. Pullback was obtained manually in the LAD artery in non-hyperaemic conditions. The RFR pullback, used in the present study, has been shown to yield similar results to other non-hyperaemic pressure ratios in guiding pullback-guided PCI strategies [13]. However, the distinction between focal and diffuse disease can be subjective as there is no strong consensus in its definition and can sometimes lead to an overestimation of the benefit of interventions [17]. Therefore, in our study, we focused on the role of pullback itself—without distinguishing between focal and diffuse disease—in predicting treatment decisions.

This study shows an even distribution of typical cardiovascular risk factors between the Pullback and Conventional groups, including a high rate of hypertension and hypercholesterolemia, with roughly 20–25% of the participants having diabetes (Table 1). This is in line with patient characteristics of previous trials, especially FAME 2, while in ISCHEMIA the prevalence of diabetes was nearly twice as high [3], [4]. Importantly, angiographic LAD disease characteristics were also largely comparable between the two cohorts, with similar distributions of diffuse versus focal disease and comparable overall LAD disease burden (Supplementary Table 1). Together, these findings suggest that the decision to perform pullback measurement was not driven by baseline clinical risk factors or anatomical vessel features, but rather by angiographic interpretation, local protocols, and operator preference.

The clinical relevance of post-PCI FFR and its relation to disease phenotypes is well established and has gained great attention in recent years [10], [18]. Our working group has placed more focus on proper physiological assessment as well, which explains the higher number of patients in the Pullback group.

As expected, in the Pullback group, the recommended treatment approach shifted away from PCI (Table 2). Not only during the initial decision-making in the cardiac catheterization laboratory, but also during the heart team discussions. Other groups, such as Nijjer et al. and Collet et al., have already shown that pre-PCI measurements can improve procedural outcomes and hold back PCI [19], [20]. The reason is that PCI can achieve better restoration of coronary artery hemodynamics in patients with focal phenotypes. These patients benefit the most in terms of angina relief and quality of life. Meanwhile, with diffuse CAD, residual ischemia and angina are more likely to remain prevalent despite percutaneous intervention with drug-eluting stents [10].

Intriguingly, we found that this distancing from PCI did not increase the surgical revascularization rates. All included patients had significant LAD disease and, as such, they could have been surgical candidates. These patients could benefit both from the revascularization of territories affected by flow-limiting stenoses and protection against new thromboembolic events. Coronary imaging studies have shown that high-risk plaques are responsible for adverse events at mid and long-term follow-up and there is evidence that these events could be reduced with preventive revascularization [21], [22], [23]. Currently, most evidence on this myocardial infarction prevention mechanism is available for CABG [24], [25]. In our study, withholding surgery could have been related to inoperability due to anatomical factors or comorbidities. Another explanation is the lack of visible flow-limiting lesions, as this may lead to deleterious competitive flow in the surgical grafts and cause graft failure [26], [27]. However, it is most likely a consequence of a lack of evidence on surgical revascularization of diffuse coronary disease and complying with recent advances and recommendations in the treatment of coronary artery disease in general. Multiple studies have demonstrated that postponing revascularization while initially focusing on improving pharmacological therapy is an acceptable and safe approach [4], [16], [28].

Our multinomial regression analysis investigated the role of pullback as a predictor of PCI or CABG, as compared to OMT, by adjusting for clinical factors that are well known to influence outcomes after surgical revascularization and, thus, have a weight on treatment decision (Table 4) [29]. In line with previous evidence, pullback emerged as the sole independent predictor of PCI deferral by steering management toward OMT. Conversely, three-vessel disease was the only robust independent predictor of referral to CABG, reflecting established guideline recommendations. In contrast to its clear role in deferring PCI, pullback had no independent impact on the decision to refer for CABG. This reflects our everyday practice, where surgical planning is driven by anatomic complexity, and underscores the lack of robust outcome data for physiology-guided CABG.

One of the key questions relates to how these diagnostic strategies translate into clinical outcomes. Although not a primary endpoint of the present study, a pullback-guided approach was associated with a higher rate of all-cause mortality at 1 year compared with the conventional strategy. Importantly, this signal was not accompanied by significant differences in major adverse cardiovascular events, myocardial infarction, stroke, or urgent revascularization, and neither cardiovascular nor non-cardiovascular mortality alone differed significantly between groups when analysed separately (Table 3, Fig. 2A and 2B).

This apparent excess in all-cause mortality should therefore be interpreted with caution. First, the absolute number of events was small (27 deaths among 842 patients), limiting the robustness of any causal inference. Second, given the retrospective and non-randomized design, residual confounding and selection bias cannot be excluded. In particular, pullback measurements may have been preferentially performed in patients with more complex coronary anatomy, greater comorbidity burden, or perceived higher procedural risk. As such, the observed mortality difference may reflect underlying patient frailty rather than a detrimental effect of the pullback-guided strategy itself.

Finally, and most importantly, we demonstrated that all-cause mortality and MACE did not differ significantly among the three treatment strategies, with comparable time-to-event curves across groups (Fig. 3a and 3b). Consistently, within the Pullback cohort, clinical outcomes were similar across treatment strategies (Supplementary Table 3), supporting the safety of pullback-guided therapeutic decision-making. Nonetheless, prospective validation is needed to define the optimal treatment allocation across different LAD disease phenotypes. To address this gap, a prospective study from our institution (COMMIT-LAD) is currently ongoing, using standardized physiological assessment to guide treatment selection in focal versus diffuse LAD disease, with 1-year MACE and patient-reported outcomes as primary endpoints [30].

An emerging strategy for the management of diffuse CAD is the use of drug-coated balloons (DCBs), with preliminary observational studies and small trials suggesting potential advantages. However, robust evidence from large randomized controlled trials is still lacking, and current guidelines do not yet support DCBs for diffuse CAD in routine practice. Future studies are warranted to clarify whether DCB-based strategies can be an additional option in in this challenging subset of patients.

### 4.1 Limitations of the study

This is a retrospective observational cohort study. Performing pullback measurement was a clinical choice and not randomized nor regulated by a study protocol. It may have been preferentially performed in patients with complex or uncertain lesions, potentially biasing associations with both decision-making and outcomes. Different operators have different preferences, changing diagnostic plans and treatment decisions. Finally, we measured only 1-year outcomes; longer-term follow-up is needed to confirm whether conservative management informed by pullback preserves event-free survival.

## 5 Conclusions

Pullback phenotyping of functionally significant LAD disease restrains percutaneous interventions and promotes pharmacological management. There is no increase in the number of surgeries performed, despite significant LAD disease and ineligibility for PCI. The only independent predictor of CABG is three-vessel disease. Although MACE rates were not different at 1 year between the treatment strategy groups, longer follow-up is needed to explore the safety of a conservative strategy for patients with flow-limiting coronary artery disease.

## CRediT authorship contribution statement

**Roberto Bova:** Writing – review & editing, Writing – original draft, Visualization, Validation, Supervision, Software, Resources, Project administration, Methodology, Investigation, Formal analysis, Data curation, Conceptualization. **Matteo Betti:** Writing – review & editing, Writing – original draft, Visualization, Validation, Supervision, Software, Resources, Project administration, Methodology, Investigation, Formal analysis, Data curation, Conceptualization. **Samuel Heuts:** Writing – review & editing, Visualization, Validation, Supervision, Methodology, Data curation, Conceptualization. **Pieter A. Vriesendorp:** Supervision, Investigation. **Alexander J.J. Ijsselmuiden:** Supervision, Investigation. **Saman Rasoul:** Supervision, Investigation. **Mustafa Ilhan:** Supervision, Investigation. **Jindra Vainer:** Supervision, Investigation. **Ralph A.L.J. Theunissen:** Supervision, Investigation. **Leo F. Veenstra:** Supervision, Investigation. **Patty Winkler:** Supervision, Investigation. **Mera Stein:** Supervision, Investigation. **Alexander Ruiters:** Supervision, Investigation. **Daniek P.J. Slegers:** . **Arnoud W.J. van ’t Hof:** Writing – review & editing, Supervision, Methodology, Investigation, Conceptualization. **Arpad Lux:** Writing – review & editing, Visualization, Validation, Supervision, Methodology, Formal analysis, Conceptualization.

## Funding

No funding involved.

## Declaration of competing interest

The authors declare that they have no known competing financial interests or personal relationships that could have appeared to influence the work reported in this paper.
