# Supplementary material for: The impact of pullback measurement on treatment decision in significant coronary artery disease: Insights from a retrospective multicentric study
Source: Int J Cardiol Heart Vasc. 2026 Feb 10;63:101887. doi: 10.1016/j.ijcha.2026.101887 (PMC12914282; doi:10.1016/j.ijcha.2026.101887)
Supplement: Supplementary Data 1 [file mmc1.docx]

|  | **No pullback (n=281)** | **Pullback (n=561)** | **p-value** |
| --- | --- | --- | --- |
| Angiographic LAD disease distribution |  |  | 0.057 |
| Diffuse disease (≥20 mm) | 51 (43%) | 161 (53%) |  |
| Focal (<20 mm) | 68 (57%) | 142 (47%) |  |
| LAD angiographic segment stenosis score^1^ | 3.35 ± 1.33 | 3.26 ± 1.25 | 0.728 |

**Supplemental Table 1. LAD anatomy in the Pullback and Conventional group.**

LAD = left anterior descending artery

^1^Defined by assigning 1 point for mild disease (<50% diameter stenosis), 2 points for moderate disease (50–70%), and 3 points for severe disease (>70%) in each LAD segment (proximal, mid, distal), yielding a total score ranging from 0 to 9. This approach was adapted from previously described CT-based segment stenosis scores and applied to invasive angiography

|  | **OMT (n=404)** | **PCI (n=254)** | **CABG (n=184)** | **p-value** |
| --- | --- | --- | --- | --- |
| Age | 69.5 (62-76) | 70 (62-76) | 67 (61-72.5) | **0.004** |
| Male sex | 312 (77%) | 207 (81%) | 156 (85%) | 0.084 |
| Diabetes | 95 (24%) | 67 (26%) | 50 (27%) | 0.546 |
| Hypertension | 291 (72%) | 187 (74%) | 137 (74%) | 0.802 |
| Hypercholesterolemia | 238 (59%) | 181 (71%) | 137 (74%) | **<0.001** |
| COPD | 39 (9.7%) | 27 (11%) | 19 (10%) | 0.915 |
| CVA | 50 (12%) | 39 (15%) | 16 (8.7%) | 0.114 |
| PAD | 42 (10%) | 30 (12%) | 9 (4.9%) | **0.040** |
| PH | 16 (5%) | 7 (4%) | 9 (7.5%) | 0.400 |
| Dyspnoea | 168 (52%) | 79 (45%) | 69 (58%) | 0.098 |
| Angina pectoris | 209 (65%) | 109 (62%) | 84 (70%) | 0.392 |
| Smoking | 74 (18%) | 57 (22%) | 51 (28%) | **0.034** |
| Prior ACS | 74 (23%) | 42 (23%) | 29 (23%) | 0.992 |
| Known CAD | 125 (49%) | 41 (39%) | 23 (32%) | **0.022** |
| 3-vessel disease | 84 (21%) | 52 (20%) | 75 (41%) | **<0.001** |
| EuroSCORE II | 1.1 (0.8-1.7) | 1 (0.8-1.6) | 1.3 (0.8-2.4) | 0.197 |
| BMI | 27.1 (24.3-30.6) | 27.8 (25.5-31) | 27.4 (24.3-30.2) | 0.061 |
| LVEF (%) | 55 (45-60) | 55 (47-55) | 55 (50-57) | 0.216 |
| Creatinine | 89 (76-104) | 89 (77-107.5) | 84 (75-97) | 0.064 |
| eGFR | 68.6 (57-80.3) | 69.9 (50.3-81.5) | 74.5 (66.9-83.8) | **0.049** |
| LDL | 2.3 (1.7-3.1) | 2.3 (1.7-3.3) | 2.4 (1.7-3.4) | 0.589 |
| HDL | 1.2 (1-1.5) | 1.1 (1-1.3) | 1.2 (0.9-1.4) | **0.012** |
| Total cholesterol | 4.2 (3.6-5.1) | 4.3 (3.5-5.4) | 4.3 (3.5-5.5) | 0.602 |
| Haemoglobin | 8.8 (8-9.5) | 8.8 (8.1-9.4) | 9 (8.2-9.5) | 0.293 |
| HbA1c | 50 (40-66) | 47 (39-52) | 44.5 (40-55) | 0.140 |

**Supplemental Table 2. Baseline demographics and clinical characteristics across the treatment strategy groups.**

ACS = acute coronary syndrome, BMI = body mass index, CABG = coronary artery bypass grafting, CAD = coronary artery disease, COPD = chronic obstructive pulmonary disease, CVA = cerebrovascular accident, eGFR = estimated glomerular filtration rate, HbA1c = glycated haemoglobin, HDL = high-density lipoprotein cholesterol, LDL = low-density lipoprotein cholesterol, LVEF = left ventricular ejection fraction, OMT = optimal medical therapy, PAD = peripheral artery disease, PCI = percutaneous coronary intervention, PH = pulmonary hypertension

|  | **OMT (n=289)** | **PCI (n=152)** | **CABG (n=120)** | **p-value** |
| --- | --- | --- | --- | --- |
| Mortality at 1 year | 13 (4.5%) | 8 (5.3%) | 3 (2.5%) | 0.517 |
| CV death | 5 (1.7%) | 2 (1.3%) | 1 (0.8%) | 0.899 |
| Non-CV death | 8 (2.8%) | 6 (3.9%) | 2 (1.7%) | 0.56 |
| MACE at 1 year | 28 (9.7%) | 22 (14%) | 13 (11%) | 0.315 |
| CV death | 5 (1.7%) | 2 (1.3%) | 1 (0.8%) | 0.899 |
| Myocardial infarction | 8 (2.8%) | 10 (6.6%) | 6 (5.0%) | 0.155 |
| Urgent revascularization | 10 (3.5%) | 8 (5.3%) | 5 (4.2%) | 0.638 |
| Stroke | 5 (1.7%) | 2 (1.3%) | 1 (0.8%) | 0.899 |

**Supplemental Table 3. Clinical outcomes in the treatment strategy groups within the Pullback cohort.**

CABG = coronary artery bypass grafting, CV = cardiovascular, MACE = major adverse cardiovascular events, OMT = optimal medical therapy, PCI = percutaneous coronary intervention
